# Estimating an exchange rate between the EQ-5D-3L and ASCOT

**DOI:** 10.1007/s10198-017-0910-x

**Published:** 2017-06-16

**Authors:** Katherine Stevens, John Brazier, Donna Rowen

**Affiliations:** School of Health and Related Research, University of Sheffield, Regent Court, 30 Regent Street, Sheffield, S1 4DA UK

**Keywords:** Preference-based methods, Utility, Social care, EQ-5D, ASCOT, I10

## Abstract

**Background:**

The aim was to estimate an exchange rate between EQ-5D-3L and the Adult Social Care Outcome Tool (ASCOT) using preference-based mapping via common time trade-off (TTO) valuations. EQ-5D and ASCOT are useful for examining cost-effectiveness within the health and social care sectors, respectively, but there is a policy need to understand overall benefits and compare across sectors to assess relative value for money. Standard statistical mapping is unsuitable since it relies on conceptual overlap of the measures but EQ-5D and ASCOT have different conceptualisations of quality of life.

**Methods:**

We use a preference-based mapping approach to estimate the exchange rate using common TTO valuations for both measures. A sample of health states from each measure was valued using TTO by 200 members of the UK adult general population. Regression analyses are used to generate separate equations between EQ-5D-3L and ASCOT values using their original value set and TTO values elicited here. These are solved as simultaneous equations to estimate the relationship between EQ-5D-3L and ASCOT.

**Results:**

The relationship for moving from ASCOT to EQ-5D-3L is a linear transformation with an intercept of −0.0488 and gradient of 0.978. This enables QALY gains generated by ASCOT and EQ-5D to be compared across different interventions.

**Conclusions:**

This paper estimated an exchange rate between ASCOT and EQ-5D-3L using a preference-based mapping approach that does not compromise the descriptive systems of the two measures. This contributes to the development of preference-based mapping through the use of TTO as the common metric used to estimate the exchange rate between measures.

## Introduction

A problem in comparing the cost-effectiveness of interventions across sectors is the use of different outcome measures. Whilst agencies such as NICE (National Institute of Health and Care Excellence) [1] advocate the use of EQ-5D for health technology assessment in the UK for reasons of comparability across interventions for different diseases and patient groups, EQ-5D may not be the most appropriate measure in evaluations of social care interventions. Although the EQ-5D [2] has become the most widely used generic measure of patient reported outcome in health care, in social care there is the increasingly used Adult Social Care Outcome Tool (ASCOT) [3]. Each measure is useful for examining cost-effectiveness within their sector but this raises the issue of comparability of evaluations undertaken in different sectors using different outcome measures to assess benefit.

The 5-year forward view for the UK National Health Service (NHS) [4], set out ambitions for a future in which networks of care are managed around the individual, and new models of care facilitate integration across acute, primary, mental, specialist and social care services. This greater integration in funding and delivery means greater policy emphasis on coordination between sectors, so there is a need to understand the comparative cost-effectiveness of different interventions that span these sectors. Therefore, there is a need to be able to assess benefits across different sectors to understand overall benefits and to make comparisons across sectors to assess relative value for money. One solution to this could be to use a common metric, such as a subjective wellbeing measure [5]; however, existing wellbeing measures such as life satisfaction scales are not designed for use in economic evaluation and there are major concerns about their validity and appropriateness in health. They have also been found to be insensitive to physical health [6]. Another solution, and the focus of this paper, is to value measures (such as the EQ-5D and ASCOT) on a common scale by estimating exchange rates between them.

The EQ-5D measures an individual’s health status across five dimensions: mobility, self-care, usual activities, pain or discomfort, and anxiety or depression. The original EQ-5D (EQ-5D-3L) contains three levels for each dimension (no problem, some problems and severe problems). A five-level version (EQ-5D-5L) is also now in use (no problem, mild problems, moderate problems, severe problems and extreme problems). The EQ-5D-3L can be converted to a UK preference-weighted index using a value set obtained from a large survey of the UK general public using a variant of time trade-off (TTO) anchored on full health at one and dead at zero [2]. The best state is valued at 1 and the worst state is valued at −0.594 [2]. A more recent study has also been undertaken to provide preference-based values for the EQ-5D-5L [7]. The index is used to generate quality adjusted life years (QALYs) for use in economic evaluations.

ASCOT is a measure of social-care quality of life that is designed to assess the extent to which an individual’s needs and wants are being met [3]. It has eight dimensions: accommodation, cleanliness and comfort, safety, food and drink, personal care, control over daily life, social participation and involvement, and dignity. Each is assessed across four levels: high unmet needs, some unmet needs, no unmet needs and ideal level achieved. The state 11111111 denotes the best state. There are two methods of scoring the instrument. One is to use scores developed from a general population survey using best-worst scaling (BWS) [3], but these values are not able to generate QALYs as they are not anchored on the 1-0 full health-dead scale required to generate QALYs. The second is to use the mapping function between BWS and TTO which was developed by valuing a sample of ASCOT states by TTO, then estimating a mapping function between BWS and TTO that generates values for all ASCOT states on the 1-0 full health-dead scale and can be used to generate QALYs [3].

Although both the EQ-5D-3L and ASCOT can be used to generate QALYs, differences in the descriptive systems and valuation methods means they are not measuring the same concepts and hence results are not directly comparable. These instruments are trying to measure different conceptualisations of life. EQ-5D-3L is about five key aspects of a person’s health, whereas ASCOT is concerned with the way a person’s health, combined with their socio-economic status, home circumstances (including availability of informal care) and the social care services they receive impacts on their overall quality of life in terms of the extent to which their needs and wants are being met. While there is moderate (0.47) correlation between the measures [8], they are not measuring the same concepts, since EQ-5D is concerned with health-related quality of life whereas ASCOT focuses upon social care needs and wants.

Although these measures have both been scored and anchored on the QALY scale using similar versions of TTO, the two TTO tasks differed in a crucial way. The upper anchor in the TTO for EQ-5D-3L was EQ-5D-3L state 11111 (no health problems) and for ASCOT was ASCOT state 11111111 (meeting all social care related needs and wants). These upper anchors are not the same and so this may result in important differences in the scales. Furthermore, the UK TTO values obtained in the original valuation of the EQ-5D-3L [2] have not been replicated in subsequent surveys [9, 10]. This suggests that values obtained more than 20 years ago may be responsible for further differences with ASCOT.

There are different approaches to enhancing comparability between the measures. One is the conventional mapping approach which involves estimating a statistical relationship between the measures, but this relies on a strong and meaningful statistical relationship, which is unlikely given the differences at the conceptual level. An alternative approach is a preference-mapping approach that converts between measures using preferences over both measures, rather than statistical association in self-reported health states. Preference-mapping has previously valued a range of measures (generic health-related quality of life measures of EQ-5D-3L, SF-6D and HUI3, social care measures OPUS (an earlier version of ASCOT [11]) and ICECAP-O and asthma-specific AQL-5D) using a generic visual analogue scale (VAS) (best imaginable to worst imaginable life) and ranking methods on a common scale [12, 13]. Regression analyses are then performed to be able to convert between the measures where the conversion is via preferences on the common VAS or ranking scale. This preference-based mapping approach is used here to generate an exchange rate between EQ-5D-3L and ASCOT using TTO (rather than VAS or ranking) to provide the common scale used to produce the exchange rate. The purpose of this study was not to replicate the EQ-5D-3L valuation, but to understand the relationship between the EQ-5D-3L and ASCOT.

## Methods

We conducted interviews using the TTO methodology to simultaneously value a subset of both EQ-5D-3L and ASCOT states. We modelled the data to estimate the exchange rate or relationship between EQ-5D-3L and ASCOT using the preference-based mapping approach that has previously been used with VAS [12] and ranking data [13].

### Selection of states

Fifty states were selected from each descriptive system (EQ-5D-3L and ASCOT) to reflect the full utility score range of each measure according to the existing value set and the severity levels of each dimension. For each descriptive system, the 50 states selected were sorted into ten blocks of five states. States were allocated to blocks to also ensure this severity range in each block. Each respondent valued a total of ten states made up from one block of five EQ-5D-3L states and one block of five ASCOT states. The order in which the blocks appeared in the interview was randomised. Blocks were also randomised across respondents. The order in which attributes appeared within a state was randomised across individuals (but not within an interview).

### Valuation methodology

The sample of states from each descriptive system (EQ-5D-3L and ASCOT) was valued using a common valuation method, conducted by the same interviewers, on the same sample of the general population.

The TTO protocol was based on the original measuring and valuing health (MVH) methods for states better than and worse than dead [2] but with a generic upper anchor for the best imaginable state defined as “the best life imaginable”. This ensures that the two descriptive systems are valued on a common scale. We adapted this approach to use TTO (rather than VAS or ranking) to make it more consistent with the NICE reference case and existing studies that use TTO. However, to be able to value both measures on the same scale the best state is not instrument specific, but described in general terms of a best imaginable life.

The TTO task began by asking respondents to consider a state. They were asked whether they thought it was better or worse than dead. Their response determined whether they were asked a better than or worse than dead version of the TTO.

For better than dead, respondents were asked to consider a choice between life A which was *t* years (*t* < 10) in the best life imaginable and life B, which was 10 years in the state being valued.

For worse than dead, respondents were asked to consider a choice between life A which was 10-*t* years in the state, followed by *t* years in the best life imaginable and life B, which was to die immediately. The value of *t* representing indifference was determined using the titration method.

### Interviews

Computer assisted personal interviews (CAPI) were undertaken by a survey company in five locations throughout England and Wales using a hall test methodology, whereby participants were recruited to a community location to undertake an interview. The survey company were highly experienced at this type of preference-based interview, having done many projects of this type before. The survey company employs professional interviewers who undertook the interviews. All locations had internet access and the interview survey was carried out online via a web link. All interviews were undertaken individually but other people may also have been undertaking the interview in another part of the hall with a different interviewer. A University of Sheffield company hosted and produced the online survey in collaboration with the research team. The hall test methodology allowed the opportunity for the lead researcher on the project to attend the project briefing and training with the interview team, answer any questions and also to observe a full day of interviews, in order to ensure quality.

Two hundred members of the adult (age 18 years or over) general population in England and Wales were surveyed. Quotas for age and gender were applied in order to obtain a representative sample. Once a respondent had consented to the interview, an interviewer set up the online survey on a laptop and then read the interview questions to the respondent. The respondent entered the responses themselves. Interviewers sat next to respondents for the duration of the interview and were able to answer any queries that arose. After completing some socio-demographic background questions, respondents completed both descriptive systems in order to familiarise themselves with them, then undertook a practice TTO question followed by 10 TTO questions. Following the interview, a thank you and £5 voucher note was issued.

### Analysis: estimating a relationship between EQ-5D-3L and ASCOT

The analysis is based on the preference-mapping approach outlined previously which was used by Rowen [12], although we used a generic TTO instead of VAS. Firstly, the relationship between the new TTO and original value sets was plotted to inform what form the model should take, for example linear, cubic or quadratic. Regression analyses were used to estimate:

1. The relationship between the new TTO values and the original value set for the EQ-5D-3L [2], and
2. The relationship between the new TTO values and the original value set for ASCOT [3].

Both mean and individual level models were considered. The root mean square error (RMSE) (of predictions at the health state level) was calculated for each model in order to compare the predictive performance of models. The models for the mean regressions and the best performing individual level models for ASCOT and EQ-5D were then solved as simultaneous equations, in order to estimate the relationship between EQ-5D-3L and ASCOT [12].

This produced a single mapping function that can convert an ASCOT utility value into the corresponding value on the utility scale of EQ-5D-3L (as used in [12]).

### Ethics

The study received ethical approval from the University of Sheffield Ethics Committee on 20/05/2015.

## Results

### Interviews and sample

Interviews were carried out in June 2015. The interviewers observed that the CAPI method worked well, respondents engaged well in the interviews and the online system was easy to operationalise. The lead researcher observed that the interview team were highly experienced and focused and conducted the interviews well.

The age, gender, education level, ethnicity, income, general health, EQ-5D and ASCOT scores of the sample are reported in Table 1, together with UK census data from 2011 (where available) for comparison

**Table 1:** Characteristics of the sample (*n* = 200)

	Sample	UK population^a^
Age (years)	Median 45 (min 18, max 84)	Median 40
Male (%)	42	49.1
Education level (%)		
Have a degree or equivalent professional qualification	32.5	27
Ethnic group (%)		
White	94	87
Mixed	1.5	2
Asian or Asian British	2.5	6
Black or Black British	1.5	3
Chinese or other ethnic group	0.5	2
Household income (%)		
Less than £9,999	10.5	
£10,000–£19,999	15.5	
£20,000–£29,999	15	
£30,000–£39,999	6	
£40,000–£49,999	8	
Greater than £50,000	9	
Would rather not say/don’t know	36	
Mean EQ-5D score	0.81	
Mean ASCOT score	0.87	
General health (%)		
Excellent	14.5	
Very good	30	
Good	32	
Fair	18.5	
Poor	5	

^a^Taken from the UK 2011 census [https://www.ons.gov.uk/peoplepopulationandcommunity/populationandmigration/populationestimates/articles/overviewoftheukpopulation/february2016

### Health state values

Descriptive statistics for all states valued are provided in Tables 2 and 3. The states are ordered by mean state value. The average number of valuations per state varied between 19 and 23 per state. The median health state value exceeded the mean in all cases. The mean health state value for the best ASCOT state was 0.93 (SD = 0.10) compared to 0.96 (0.07) for the EQ-5D-3L, though the gap between the best and mildest impaired state was 0.02 on ASCOT compared to 0.18 for EQ-5D-3L. The worst state had a value of −0.28 for ASCOT and −0.51 for the EQ-5D-3L.

**Table 2:** Health state values for ASCOT (ordered by mean value)

Health state	Mean	Median	SD	*N*
11111111	0.93	0.98	0.10	23
11212111	0.91	0.98	0.23	19
11122121	0.87	0.95	0.19	20
11212221	0.85	0.90	0.16	19
12122232	0.85	0.90	0.16	23
11131212	0.80	0.95	0.36	19
11121111	0.76	0.88	0.30	19
32132411	0.75	0.90	0.30	19
21123141	0.73	0.88	0.48	19
11222212	0.72	0.85	0.36	20
32132221	0.71	0.88	0.37	20
22221141	0.70	0.73	0.27	20
12121211	0.68	0.91	0.48	20
41213321	0.64	0.75	0.38	21
12322212	0.64	0.68	0.39	19
13232223	0.63	0.73	0.35	19
24211142	0.63	0.88	0.57	20
21431231	0.62	0.70	0.46	19
42321323	0.60	0.65	0.40	19
24121142	0.60	0.73	0.50	23
22221144	0.60	0.68	0.32	20
32312414	0.59	0.65	0.45	19
22323144	0.58	0.68	0.35	21
13122233	0.58	0.65	0.34	19
21141143	0.57	0.66	0.44	20
14112232	0.57	0.70	0.46	19
32322322	0.53	0.73	0.53	20
13232432	0.53	0.63	0.59	19
14212232	0.52	0.79	0.55	20
14422232	0.47	0.60	0.53	20
32223414	0.47	0.73	0.60	23
43132322	0.43	0.63	0.62	19
32424414	0.42	0.41	0.44	20
44123323	0.42	0.50	0.52	21
24342141	0.40	0.49	0.46	20
43322213	0.40	0.51	0.56	20
44322323	0.36	0.50	0.69	19
44242323	0.34	0.50	0.59	23
42233321	0.31	0.63	0.75	20
43121323	0.30	0.50	0.64	20
42243322	0.19	0.50	0.67	19
34444432	0.19	0.20	0.61	19
32224414	0.18	0.66	0.79	20
43333444	0.13	0.38	0.62	21
33333343	0.12	0.33	0.72	19
44444444	0.05	−0.17	0.71	20
32343444	0.01	0.18	0.63	19
33343434	−0.04	0.26	0.69	20
44344431	−0.13	−0.38	0.59	20
43232434	−0.28	−0.39	0.58	20

**Table 3:** Health state values for EQ-5D-3L (ordered by mean value)

Health state	Mean	Median	SD	*N*
11111	0.96	0.98	0.07	23
12112	0.78	0.88	0.23	19
12111	0.76	0.93	0.36	19
21112	0.72	0.73	0.31	19
11222	0.69	0.81	0.44	20
22212	0.66	0.88	0.46	19
22211	0.64	0.81	0.43	20
12213	0.63	0.70	0.40	23
21113	0.61	0.83	0.44	19
13111	0.59	0.74	0.44	20
23111	0.57	0.71	0.47	20
22123	0.55	0.65	0.47	19
11223	0.55	0.73	0.42	19
21223	0.51	0.60	0.54	19
23112	0.50	0.61	0.53	20
22221	0.49	0.58	0.47	20
12321	0.49	0.56	0.46	20
22312	0.49	0.58	0.46	21
13211	0.48	0.69	0.55	20
22222	0.44	0.50	0.54	21
23222	0.43	0.65	0.52	23
21231	0.36	0.50	0.54	21
11131	0.34	0.63	0.62	19
32221	0.33	0.50	0.52	23
12323	0.29	0.48	0.57	21
31121	0.26	0.50	0.65	19
13322	0.26	0.50	0.67	20
22331	0.23	0.48	0.60	23
31311	0.22	0.50	0.68	19
11231	0.22	0.48	0.70	19
11331	0.19	0.40	0.61	20
23123	0.13	0.44	0.68	20
33211	0.12	0.39	0.71	20
21132	0.11	0.33	0.71	19
21131	0.10	0.45	0.74	20
23312	0.09	0.14	0.60	20
21331	0.07	0.36	0.64	20
22231	0.05	−0.02	0.64	19
13323	0.03	0.20	0.78	19
31322	0.03	0.20	0.65	19
23323	0.03	0.03	0.72	19
31131	−0.08	−0.13	0.70	19
21332	−0.08	−0.02	0.57	19
31333	−0.09	−0.17	0.61	21
22132	−0.11	−0.15	0.61	20
13233	−0.14	−0.38	0.55	19
32223	−0.20	−0.47	0.67	20
31133	−0.21	−0.40	0.63	20
31323	−0.30	−0.45	0.58	19
33333	−0.51	−0.63	0.42	20

**Fig. 1:**
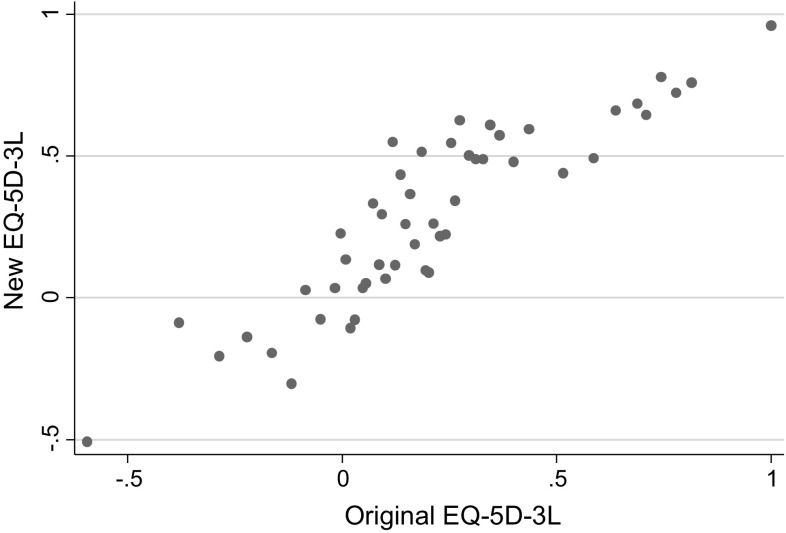
Scatter plot of original and new EQ-5D-3L values

**Fig. 2:**
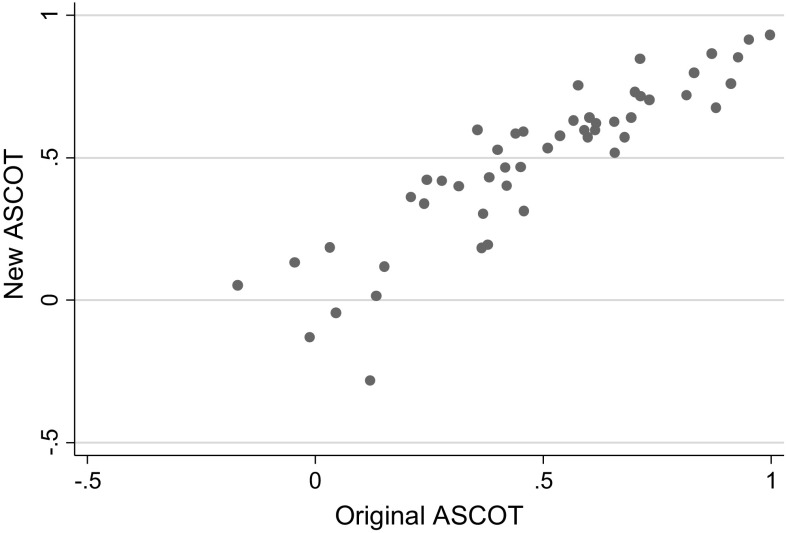
Scatter plot of original and new ASCOT values

A scatter plot of the new and original EQ-5D-3L values is presented in Fig. 1 and a scatter plot of the new and original ASCOT values is presented in Fig. 2.

### Regression results

The relationship between the new and original values for EQ-5D-3L was clearly linear (Fig. 1) and this was also true for ASCOT (Fig. 2). Mean level OLS, individual level OLS and random effects regression was used to estimate the relationships between these (new TTO and original value set per measure).

The results of these regressions for the EQ-5D-3L are shown in Table 4 and for ASCOT in Table 5.

**Table 4:** Regressions for EQ-5D-3L

	Coefficient	Standard error	*t*	*P* > $\left| t \right|$	RMSE	*n*
Mean level OLS						
Original EQ-5D-3L Utility	0.916	0.065	14.10	0.000	0.143	50
Constant	0.094	0.024	3.87	0.000		
Individual level OLS						
Original EQ-5D-3L Utility	0.911	0.057	15.91	0.000	0.079	999
Constant	0.099	0.022	4.57	0.000		

	Coefficient	Standard error	*z*	*P* > $\left| z \right|$	RMSE	*n*
Random effects GLS						
Original EQ-5D-3L Utility	0.912	0.042	21.63	0.000	0.079	999
Constant	0.099	0.032	3.10	0.002		

*OLS* ordinary least squares, *GLS* generalised least squares

**Table 5:** Regressions for ASCOT

	Coefficient	Standard error	*t*	*P* > $\left| t \right|$	RMSE	*n*
Mean level OLS						
Original ASCOT utility	0.884	0.063	14.12	0.000	0.124	50
Constant	0.057	0.035	1.63	0.109		
Individual level OLS						
Original ASCOT utility	0.887	0.057	15.49	0.000	0.031	979
Constant	0.059	0.032	1.84	0.067		

	Coefficient	Standard error	*z*	*P* > $\left| z \right|$	RMSE	*n*
Random effects GLS						
Original ASCOT utility	0.892	0.042	21.46	0.000	0.030	979
Constant	0.054	0.034	1.57	0.116		

*OLS* ordinary least squares, *GLS* generalised least squares

For both mean models the adjusted R-squared exceeded 0.8. The results from these models gave equations for predicting new utility values from the original ones (for both the EQ-5D-3L and ASCOT). These equations were then solved simultaneously to give the relationship between ASCOT and EQ-5D-3L as shown below.

1 $$( {\text{a)}}\;{\text{New generic TTO utility }} = \, 0.0 9 4+ \, \left( {0. 9 1 6*{\text{EQ-}}5 {\text{D-}}3 {\text{L original value}}} \right)$$

2 $$( {\text{b)}}\;{\text{New generic TTO utility }} = \, 0.0 5 7+ \, \left( {0. 8 8 4 { }*{\text{ASCOT original value}}} \right)$$

Solving Eqs. (1) and (2) as simultaneous equations:

3 $$0.0 5 7 4 3 3 7+ \, \left( {0. 8 8 4 { }*{\text{ASCOT}}} \right) \, = 0.0 9 4+ \, \left( {0. 9 1 6*{\text{EQ-}}5 {\text{D-}}3 {\text{L}}} \right)$$

EQ-5D-3L = −0.040 + (0.965*ASCOT).

For the EQ-5D-3L models, the RMSE was the same for the individual level OLS model and the random effects GLS model (0.079). For the ASCOT models, the random effects GLS model had a lower RMSE (0.03) than the individual level OLS model (0.031). The random effects GLS models have the lowest RMSE, and so are the preferred models estimated using individual level data, and are preferred to the mean level models. The individual level random effects GLS models were solved simultaneously to give the relationship between ASCOT and EQ-5D-3L as follows:

4 $$\left( {\text{a}} \right){\text{ New generic TTO utility }} = \, 0.0 9 9+ \, \left( {0. 9 1 2*{\text{EQ-}}5 {\text{D-}}3 {\text{L original value}}} \right)$$

5 $$\left( {\text{b}} \right){\text{ New generic TTO utility }} = \, 0.0 5 4+ \, \left( {0. 8 9 2 { }*{\text{ASCOT original value}}} \right)$$

Solving Eqs. (4) and (5) as simultaneous equations:

$$0.0 5 40 2 3 4+ \, \left( {0. 8 9 2 4 5 6 4 { }*{\text{ASCOT}}} \right) \, = 0.0 9 8 5 7 7 1+ \, \left( {0. 9 1 2 4 9 3 2*{\text{EQ-}}5 {\text{D-}}3 {\text{L}}} \right)$$

EQ-5D-3L = −0.04883 + (0.978042*ASCOT). Table 6 shows some examples of ASCOT values and their corresponding EQ-5D-3L values using this equation. EQ-5D-3L values are consistently lower than ASCOT, but the differences are always less than 0.1. The largest differences are at the upper end, where EQ-5D-3L values are 0.08 lower than ASCOT at 1.0, and 0.07 at 0.8.

**Table 6:** Example transformations

ASCOT	EQ-5D-3L using mean model	EQ-5D-3L using random effects model
1	0.92	0.93
0.8	0.73	0.73
0.6	0.54	0.54
0.4	0.35	0.34
0.2	0.15	0.15
0.0	−0.04	−0.05
−0.2	−0.23	−0.24

## Discussion

This paper estimated an exchange rate between ASCOT and EQ-5D-3L using a preference-based approach that does not compromise the descriptive systems of the two measures. It applied a preference-based mapping approach [12] using a generic version of TTO to value each instrument.

The method of recruiting and interviewing worked well. The advantages of a hall test methodology are that a member of the research team was able to observe all interviews for a day and so was able to ensure the quality of the interviews. They were also able to brief the interviewers and answer any questions. Furthermore, a supervisor was present at each location the interviews were carried out in and due to the use of CAPI techniques, this ensured the script was adhered to.

The sample was broadly representative of the UK general population although the median age was higher (45 versus 40), there were fewer males (42 versus 49.1%) and a slightly higher percentage of respondents had a first degree or equivalent (32.5 versus 27%). There was also a larger proportion of white participants (94 versus 87%).

This paper contributes to the development of preference-based mapping through the use of TTO as the common metric used to estimate the exchange rate between the measures. Previous studies used VAS [12] and ranking [13], which are techniques less commonly used to value measures since they do not ask participants to make a trade-off when assessing health states. The advantage of using TTO as the common metric is that it is a commonly used technique which has been used in many studies (including EQ-5D-3L, ASCOT and EQ-5D-5L [7]) that involves the use of trade-offs between quality of life and length of life to assess the value of different health states.

The advantage of this preference-based mapping approach is that it enables the comparison of evaluations using EQ-5D-3L and ASCOT to enable comparison of the benefits of interventions across both health and social care sectors. Unlike standard statistical mapping it does not rely on the overlap of measures administered at the same time to the same person, since the measures have different conceptualisations of life and may not be typically administered to the same groups of people. In addition, it does not simply compare between the value sets of the measures, which are likely to differ due to their different valuation protocols. The preferred exchange rate could be used to convert, say, mean ASCOT values of 0.4 and 0.8 into EQ-5D values of 0.34 and 0.73, respectively, using Table 6.

The random effects GLS models estimated using individual level data are the preferred models. These are solved as simultaneous equations to generate the exchange rate between ASCOT and EQ-5D-3L. The relationship between the measures is a linear transformation with an intercept of −0.049 and gradient of 0.978 for moving from ASCOT to EQ-5D-3L. The differences between the original scales of the instruments are never larger than 0.1. The differences between the intervals (e.g. 0.8–1.0) are less, with, for example, a move of 0.2 on the ASCOT being between 0.19 and 0.2 on EQ-5D-3L. This would suggest that for many interventions QALY gain generated by ASCOT will be comparable to that for the EQ-5D-3L. However, this does not suggest that an individual completing the EQ-5D-3L and ASCOT would give comparable values since this is also driven by the descriptive system. It means that given the initial selection of measure, values on the ASCOT can be converted into EQ-5D-3L currency using the linear transformation equation. For example, a person completing both the EQ-5D and ASCOT classification systems will be reporting different aspects of quality of life using each descriptive system, and these will then be scored using the existing value sets for each measure. The corresponding utility score that is generated for each measure may be quite different, and equally their change in utility when responding at a second time point may also differ. For example, their mobility may change in EQ-5D yet this may impact on their control over daily life, social participation and involvement and dignity in ASCOT, meaning that the change in utility will be different. The mapping algorithm reported here enables both EQ-5D and ASCOT QALY estimates to be compared, thus enabling more consistent decision making when making resource allocation decisions across different sectors.

The mean and median values for the best state for each measure are below 1 (though they are very close to 1) which may be particularly surprising for ASCOT where each dimension is in their ‘ideal state’. However, as the upper anchor used in the TTO task was “the best life imaginable” it is understandable that respondents may not feel that the measure-specific best state (i.e. state 11111 for EQ-5D and state 11111111 for ASCOT) describe the best life imaginable.

The model specifications include utility values as explanatory variables rather than dimension levels, as it is expected that the exchange rate will be applied to observed utility values rather than health states. This means that the exchange rate can be applied to any data where mean values are available.

A limitation of the study is that whilst the relationship between EQ-5D new TTO and the original value set is linear, it is true that the new values are slightly higher, which may cast doubt on the accuracy of the new TTO results. However, this may be expected since the upper anchor is different, the studies were conducted many years apart and the original valuation was paper-based whereas the new study was CAPI.

Another limitation is that the application of the results is focussed on UK usage. The models reported here use the UK value sets of EQ-5D and ASCOT that represent UK preferences. Although value sets for many countries exist for EQ-5D, ASCOT has only a UK value set. If ASCOT value sets are made available for other countries the data used here can be remodelled to generate an exchange rate between ASCOT and EQ-5D values for those other countries.

## References

[1] National Institute for Health and Care Excellence. Guide to the methods of technology appraisal. https://www.nice.org.uk/article/pmg9/resources/non-guidance-guide-to-the-methods-of-technology-appraisal-2013-pdf (2013). Accessed 14 Dec 2015 27905712

[2] Dolan P (1997). Modeling valuations for EuroQol health. Med. Care.

[3] Netten A, Burge P, Malley J (2012). Outcomes of social care for adults: developing a preference-weighted measure. Health Technol. Assess..

[4] NHS England: Five Year Forward View. https://www.england.nhs.uk/wp-content/uploads/2014/10/5yfv-web.pdf (2014). Accessed 12 June 2017

[5] O’Donnell G, Deaton A, Durand M, Halpern D, Layard R (2014). Wellbeing and policy.

[6] Mukuria C, Rowen D, Peasgood T, Brazier J.: An empirical comparison of wellbeing measures used in the UK. EEPRU Report 027.7 (2015)

[7] Devlin N, Shah K, Feng Y, Mulhern B, van Hout B.: An EQ-5D-5L value set for England. HEDS discussion paper series 16/02. University of Sheffield (2016)

[8] Mukuria C, Peasgood T, Rowen D, Brazier J.: An empirical comparison of well-being measures used in UK. EEPRU Research Report RR0048 (2016)

[9] Tsuchiya A, Brazier JE, Roberts J (2006). Comparison of valuation methods used to generate the EQ5D and the SF6D value sets in the UK. J. Health Econ..

[10] Longworth L, Yang Y, Young T, Mulhern B (2014). Use of generic and condition specific measures of health related quality of life in NICE decision making. Health Technol. Assess..

[11] Netten A, Ryan M, Smith P et al.: The development of a measure of social care outcome for older people. PSSRU discussion paper. 1690/2 (2002)

[12] Rowen D, Brazier J, Tsuchiya A, Hernandez Alava M (2012). Valuing states from multiple measures on the same VAS: a feasibility study. Health Econ..

[13] Alava MH, Brazier J, Rowen D, Tsuchiya A (2013). Common scale valuations across preference-based measures: estimation using rank data. Med. Decis. Making.

